# P-2332. Pre to Post *COVID-19* pandemic: Analyzing Recovery of Respiratory Viral Circulation Patterns

**DOI:** 10.1093/ofid/ofae631.2484

**Published:** 2025-01-29

**Authors:** Kim El Haddad, Wei Liu, Patrick Burke, Frank Esper

**Affiliations:** Cleveland Clinic Children's, Cleveland, Ohio; Cleveland Clinic, Cleveland, OH; The Cleveland Clinic, Cleveland, Ohio; Cleveland Clinic Children's, Cleveland, Ohio

## Abstract

**Background:**

The COVID-19 pandemic and resulting worldwide countermeasures immediately impacted the circulation of endemic respiratory viruses. Yet the dynamics of recovery have varied between species. We hypothesize the level of disruption in virus circulation and the time to return to pre-pandemic norms is dependent on circulation overlap with the pandemic strain.

**Figure:**
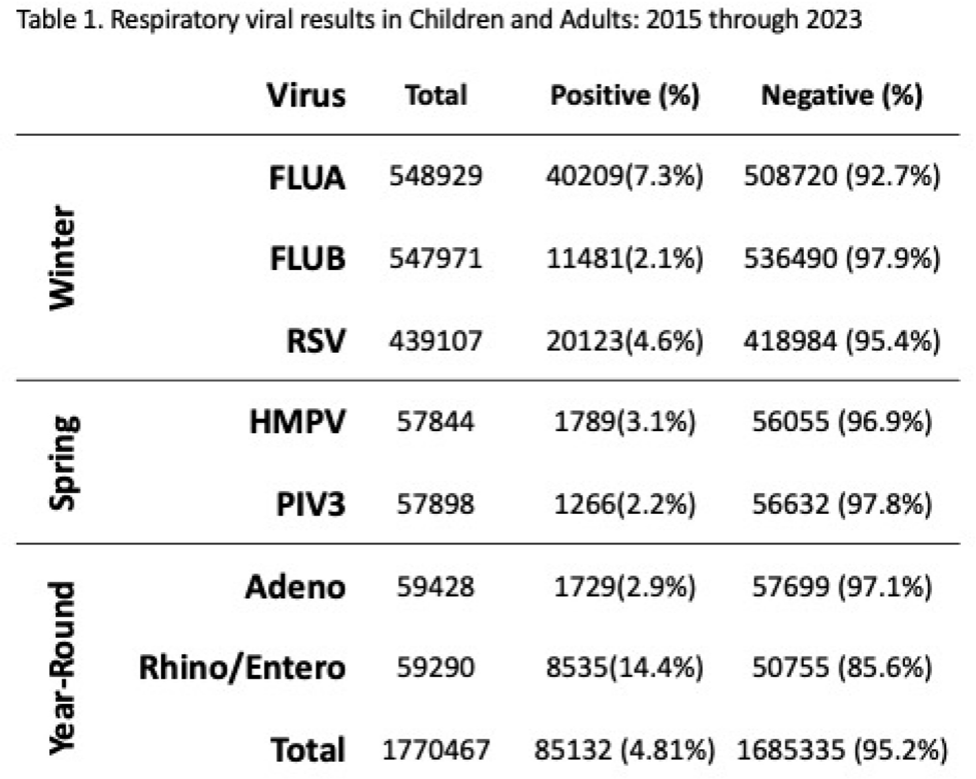

**Methods:**

Interrupted time series (ITS) analysis and continuous wavelet analysis of univariate times series (Morlet) was conducted on Cleveland Clinic respiratory virus results from 2015 through 2023. Data was analyzed on individual virus species grouped in 3 seasonality patterns: wintertime (FLUA, FLUB, RSV), springtime (HMPV, PIV3) and year-round (ADENO, RHINO/ENTERO*)*. Pre and post pandemic trends in positivity, peak shift analysis and periodic cycle were assessed.

**Figure:**
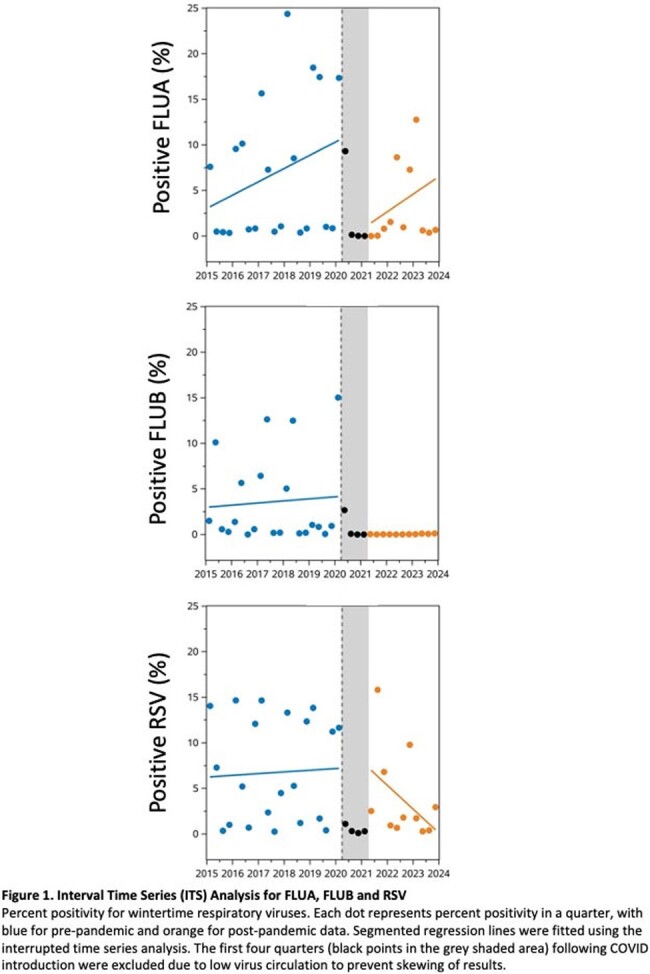

**Results:**

FLUA positivity dropped by 11% (95% CI 7.4-14%) in first effective quarter post-pandemic and gradually increased (+1.9% per year, p=.046) afterwards. The dominant cycle shifted from annual pre-pandemic to semiannual, with virus incidence peaks (median week) at 21-W50, 22-W17, and 22-W51, respectively, departing from the pre-pandemic baseline (W7, p < 0.001). RSV positivity decreased by 2.6% per year after the pandemic (95% CI 1.2-4.0%). The annual cycle remained dominant, but peak incidence of RSV shifted earlier then gradually normalized (baseline-W1 vs. 21-W39 vs. 23-W45, p< .0001). FLUB positivity significantly decreased postpandemic (0.18% vs 6.1%, p< .0001). No significant impact on PIV3 and HMPV positivity occurred. Both viruses showed an annual cycle dominance throughout, with a semiannual cycle only observed post-pandemic. ADENO showed decreasing trends before the pandemic and increasing trends after the pandemic (-0.63% vs +0.94%, p=.001). Both ADENO and RHINO/ENTERO did not show changes in circulation with no distinct peaks observed.

**Figure:**
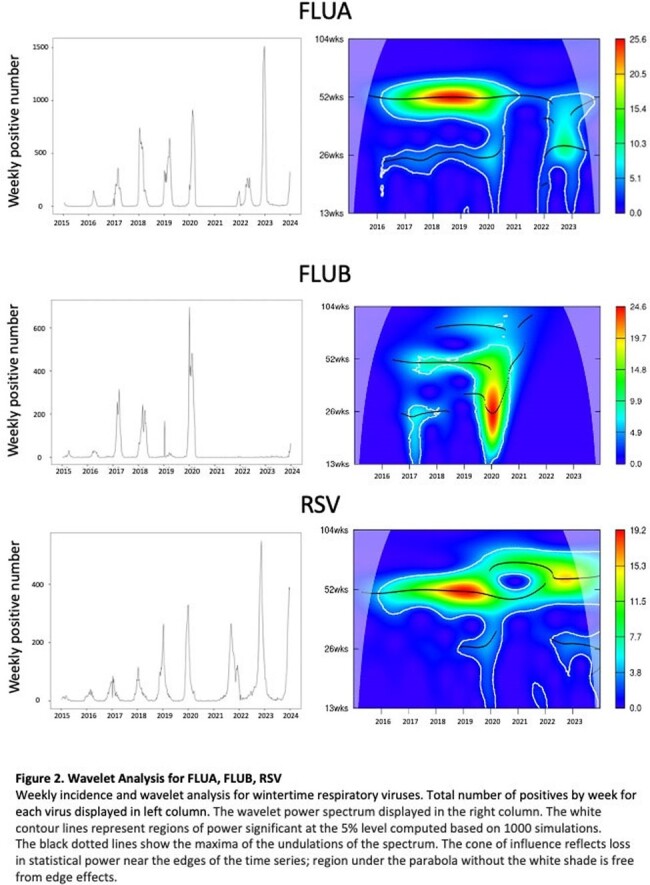

**Conclusion:**

Pandemic circulation disproportionately affects co-circulating viruses. Wintertime dominant viruses experienced prolonged displacement and have yet to return to pre-pandemic norms. Conversely, spring and year-round viruses were only modestly displaced and are now near pre-pandemic baseline. This study holds important implications for public health preparedness for future pandemics.

**Figure:**
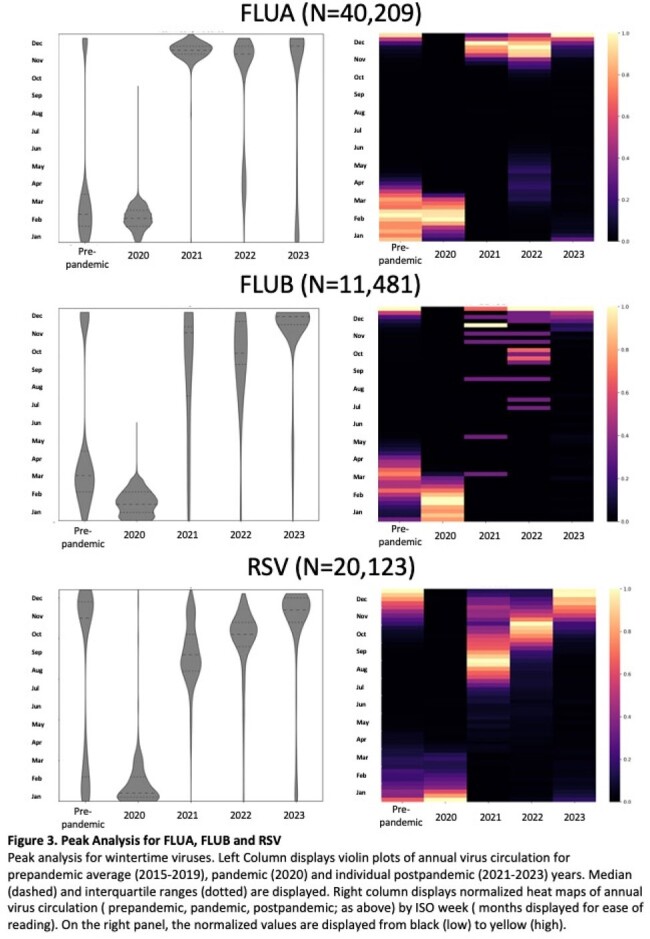

**Disclosures:**

All Authors: No reported disclosures

